# Frataxin controls ketone body metabolism through regulation of OXCT1

**DOI:** 10.1093/pnasnexus/pgac142

**Published:** 2022-07-26

**Authors:** Yi NA Dong, Clementina Mesaros, Peining Xu, Elizabeth Mercado-Ayón, Sarah Halawani, Lucie Vanessa Ngaba, Nathan Warren, Patrick Sleiman, Layne N Rodden, Kimberly A Schadt, Ian A Blair, David R Lynch

**Affiliations:** Departments of Pediatrics and Neurology, The Children's Hospital of Philadelphia, Philadelphia, PA 19104, USA; Center of Excellence in Environmental Toxicology, Department of Systems Pharmacology and Translational Therapeutics, Perelman School of Medicine, University of Pennsylvania, Philadelphia, PA 19104, USA; Center of Excellence in Environmental Toxicology, Department of Systems Pharmacology and Translational Therapeutics, Perelman School of Medicine, University of Pennsylvania, Philadelphia, PA 19104, USA; Perelman School of Medicine, University of Pennsylvania, Philadelphia, PA 19104, USA; Departments of Pediatrics and Neurology, The Children's Hospital of Philadelphia, Philadelphia, PA 19104, USA; Departments of Pediatrics and Neurology, The Children's Hospital of Philadelphia, Philadelphia, PA 19104, USA; Departments of Pediatrics and Neurology, The Children's Hospital of Philadelphia, Philadelphia, PA 19104, USA; Departments of Pediatrics and Neurology, The Children's Hospital of Philadelphia, Philadelphia, PA 19104, USA; Departments of Pediatrics and Neurology, The Children's Hospital of Philadelphia, Philadelphia, PA 19104, USA; Departments of Pediatrics and Neurology, The Children's Hospital of Philadelphia, Philadelphia, PA 19104, USA; Center of Excellence in Environmental Toxicology, Department of Systems Pharmacology and Translational Therapeutics, Perelman School of Medicine, University of Pennsylvania, Philadelphia, PA 19104, USA; Perelman School of Medicine, University of Pennsylvania, Philadelphia, PA 19104, USA; Departments of Pediatrics and Neurology, The Children's Hospital of Philadelphia, Philadelphia, PA 19104, USA; Perelman School of Medicine, University of Pennsylvania, Philadelphia, PA 19104, USA

**Keywords:** frataxin, OXCT1, ketone body, Friedreich's ataxia

## Abstract

Friedreich’s ataxia (FRDA) is an autosomal recessive neurodegenerative disease caused by the deficiency of mitochondrial protein frataxin, which plays a crucial role in iron–sulphur cluster formation and ATP production. The cellular function of frataxin is not entirely known. Here, we demonstrate that frataxin controls ketone body metabolism through regulation of 3-Oxoacid CoA-Transferase 1 (OXCT1), a rate limiting enzyme catalyzing the conversion of ketone bodies to acetoacetyl-CoA that is then fed into the Krebs cycle. Biochemical studies show a physical interaction between frataxin and OXCT1 both *in vivo* and *in vitro*. Frataxin overexpression also increases OXCT1 protein levels in human skin fibroblasts while frataxin deficiency decreases OXCT1 in multiple cell types including cerebellum and skeletal muscle both acutely and chronically, suggesting that frataxin directly regulates OXCT1. This regulation is mediated by frataxin-dependent suppression of ubiquitin–proteasome system (UPS)-dependent OXCT1 degradation. Concomitantly, plasma ketone bodies are significantly elevated in frataxin deficient knock-in/knockout (KIKO) mice with no change in the levels of other enzymes involved in ketone body production. In addition, ketone bodies fail to be metabolized to acetyl-CoA accompanied by increased succinyl-CoA *in vitro* in frataxin deficient cells, suggesting that ketone body elevation is caused by frataxin-dependent reduction of OXCT1 leading to deficits in tissue utilization of ketone bodies. Considering the potential role of metabolic abnormalities and deficiency of ATP production in FRDA, our results suggest a new role for frataxin in ketone body metabolism and also suggest modulation of OXCT1 may be a potential therapeutic approach for FRDA.

Significance Statement Understanding the function of frataxin is critical for developing treatments for Friedreich's ataxia, which currently lacks a proven therapy. The present study demonstrates a new role of frataxin in ketone body metabolism through regulation of 3-Oxoacid CoA-Transferase 1 (OXCT1). As an alternative energy source at low glucose levels, ketone body utilization deficits triggered by OXCT1 reduction can lead to failure in ATP production and oxidative stress in tissues like skeletal muscle and cerebellum, contributing to disease progression. The study provides a new method to understand the clinical features of Friedreich’s ataxia (FRDA) and identifies OXCT1 as a potential therapeutic target for FRDA.

## Introduction

Friedreich's ataxia (FRDA) is an autosomal recessive neurodegenerative disease caused by the deficiency of mitochondrial protein frataxin. The primary symptoms of FRDA include progressive ataxia, a hypertrophic cardiomyopathy, scoliosis, and in some individuals diabetes (1). A total of 96% of mutations consist of expanded guanine–adenine–adenine (GAA) repeats; such repeats partially silence the *FXN* gene and markedly decrease levels of frataxin protein (2). The remaining mutations include point mutations and deletions, all of which decrease functional frataxin (3). The age of onset correlates with shorter GAA repeat length and levels of frataxin in peripheral tissue (4).

Frataxin is a small mitochondrial protein (210 amino acids) crucial for the formation of iron–sulfur clusters for proteins including the mitochondrial enzymes aconitase and respiratory chain complexes I and II, the deficiency of which leads to diminished ATP production, mitochondrial dysfunction, and possible sensitivity to reactive oxygen species (5–9). These processes may play roles in the pathophysiology of FRDA, but the role of mitochondria in metabolism suggests metabolic events may also mediate some components of the disease, either in overall progression or in components such as diabetes. For example, platelets from patients with FRDA utilize fatty acids in preference to glucose in bypassing disabled Krebs cycle enzymes (10), suggestive of generalized metabolic dysfunction in FRDA. Moreover, FRDA patients possess exercise intolerance and abnormal post exercise recovery (11, 12), suggesting that altered metabolism might play a role in the symptomatology of FRDA.

3-Oxoacid CoA-Transferase 1 (OXCT1) is the rate-limiting enzyme converting extrahepatic ketone bodies to acetoacetyl-CoA that is fed into the TCA cycle for ATP production. Ketone bodies provide an alternative energy source to glucose for heart, brain, and skeletal muscle during times of low glucose levels such as fasting and prolonged exercise. Germline OXCT1-knockout (KO) mice, which lack the ability to oxidize ketone bodies in any tissue, exhibit lethality within 48 h of birth due to hyperketonemic hypoglycemia (13). Patients with OXCT1 deficiency, caused by mutations in the *OXCT1* gene, present with episode of ketoacidosis (build-up of ketone bodies in the body) (14–16). In the current study, we have identified a biochemical interaction of frataxin with OXCT1 in cells including neurons. In patients with FRDA and in models of frataxin deficiency, OXCT1 levels decline with disease, and associate with deficiency of ketone body metabolism both *in vivo* and *in vitro*. Such findings may provide a novel method for understanding clinical and basic features of FRDA.

## Results

### OXCT1 physically interacts with frataxin both *in vivo* and *in vitro*

**Figure 1.**
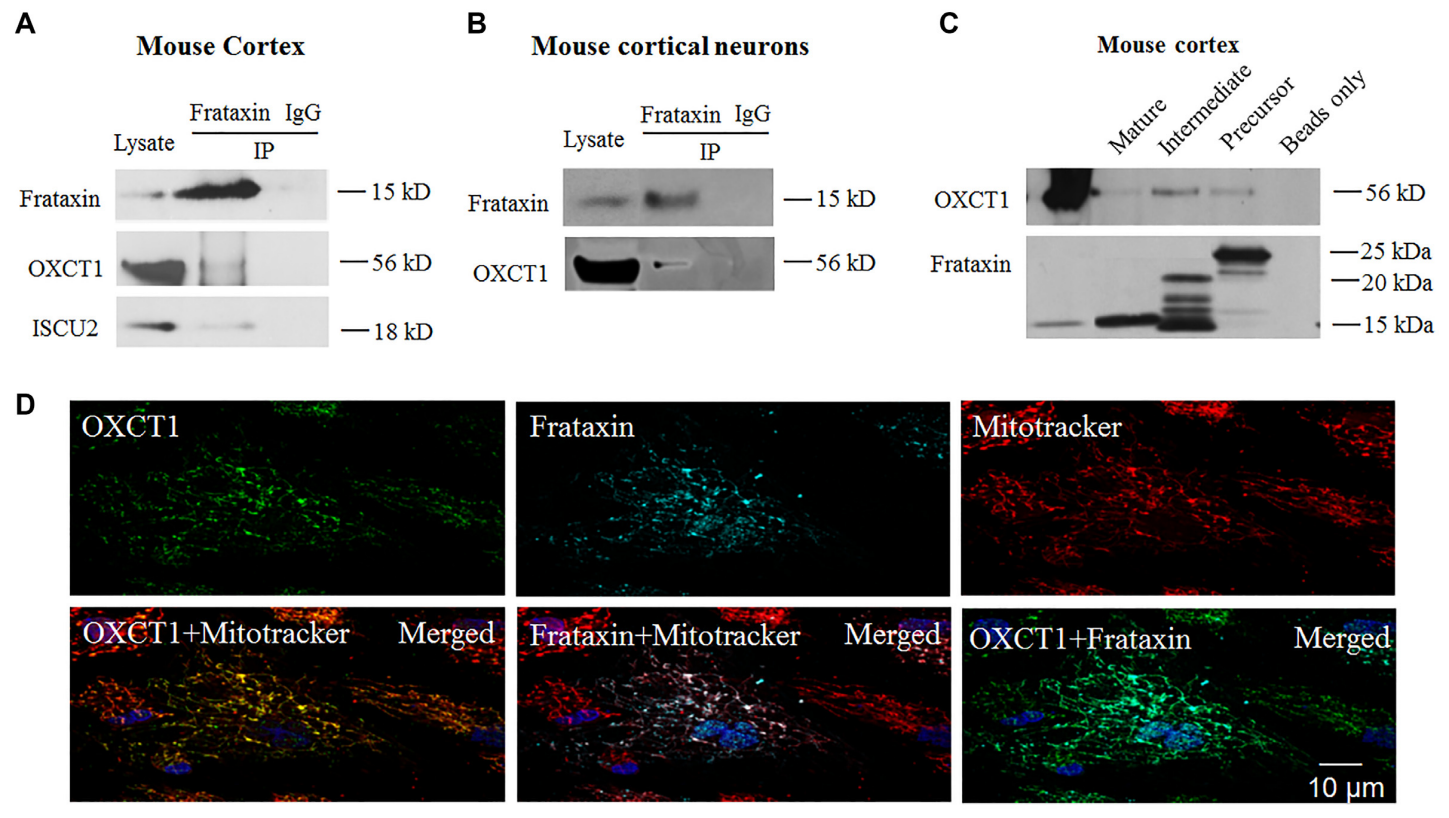
OXCT1 physically interacts with frataxin both *in vivo* in mouse cortex and *in vitro* in cortical neurons. Frataxin but not control IgG immunoprecipitated OXCT1 from both cortical homogenates (A) and neuronal lysates (B). ISCU2 was used as a positive control (A). Similarly, purified human frataxin precursor (6XHis-frataxin^1–210^), intermediate (6XHis-frataxin^42–210^), and mature form (frataxin^81–210^-6XHis) pulled down OXCT1 from cortical homogenate. Frataxin omitted (beads only) control showed no immunoreactivity (C). In human skin fibroblasts transduced with pHAGE-frataxin (D), OXCT1 colocalized with frataxin. Both proteins also colocalized with mitotracker, suggesting the mitochondrial localization of both OXCT1 and frataxin.

To search for frataxin binding partners in the brain, we immunoprecipitated frataxin with an anti-frataxin antibody in mouse cortical homogenates followed by mass spectrometry analysis. This approach identified OXCT1 as a potential frataxin binding partner with seven peptide fragments precipitated (Table S1). Co-immunoprecipitation in mouse cortical homogenates confirmed this interaction as anti-frataxin but not control IgG precipitated OXCT1 protein (Fig. 1A). Mitochondrial protein ISCU2 was used as a positive control (Fig. 1A). Similar result was found in cultured mouse cortical neurons (Fig. 1B), suggesting a physical interaction of OXCT1 with frataxin. We then performed a pulldown assay in mouse cortical homogenates using purified human frataxin precursor (6XHis-frataxin^1–210^), intermediate (6XHis-frataxin^42–210^), and mature form (frataxin^81–210^-6XHis). All three forms pulled down OXCT1 with the mature form pulling down OXCT1 to a lesser extent. No immunoreactivity was detected when frataxin was omitted (beads only; Fig. 1C). These results demonstrate physical interactions between OXCT1 and frataxin both *in vivo* (in mouse cortex) and *in vitro* (in mouse cortical neurons).

We next examined whether OXCT1 colocalizes with frataxin using immunofluorescence. Human skin fibroblasts transduced with lentivirus carrying pHAGE-*FXN* gene revealed punctate staining for both frataxin and OXCT1 as well as the colocalization of frataxin and OXCT1 with mitotracker (Fig. 1D), suggesting the mitochondrial localization of these two proteins. OXCT1 also colocalizes with frataxin in human skin fibroblasts (Pearson’s correlation coefficient: r = 0.659 pixel by pixel correlation) (Fig. 1D), further supporting the physical interaction of these two proteins.

### Frataxin regulates OXCT1 protein levels *in vitro* in human skin fibroblasts and *in vivo* in the cerebellum and skeletal muscle of frataxin knockdown mice

**Figure 2.**
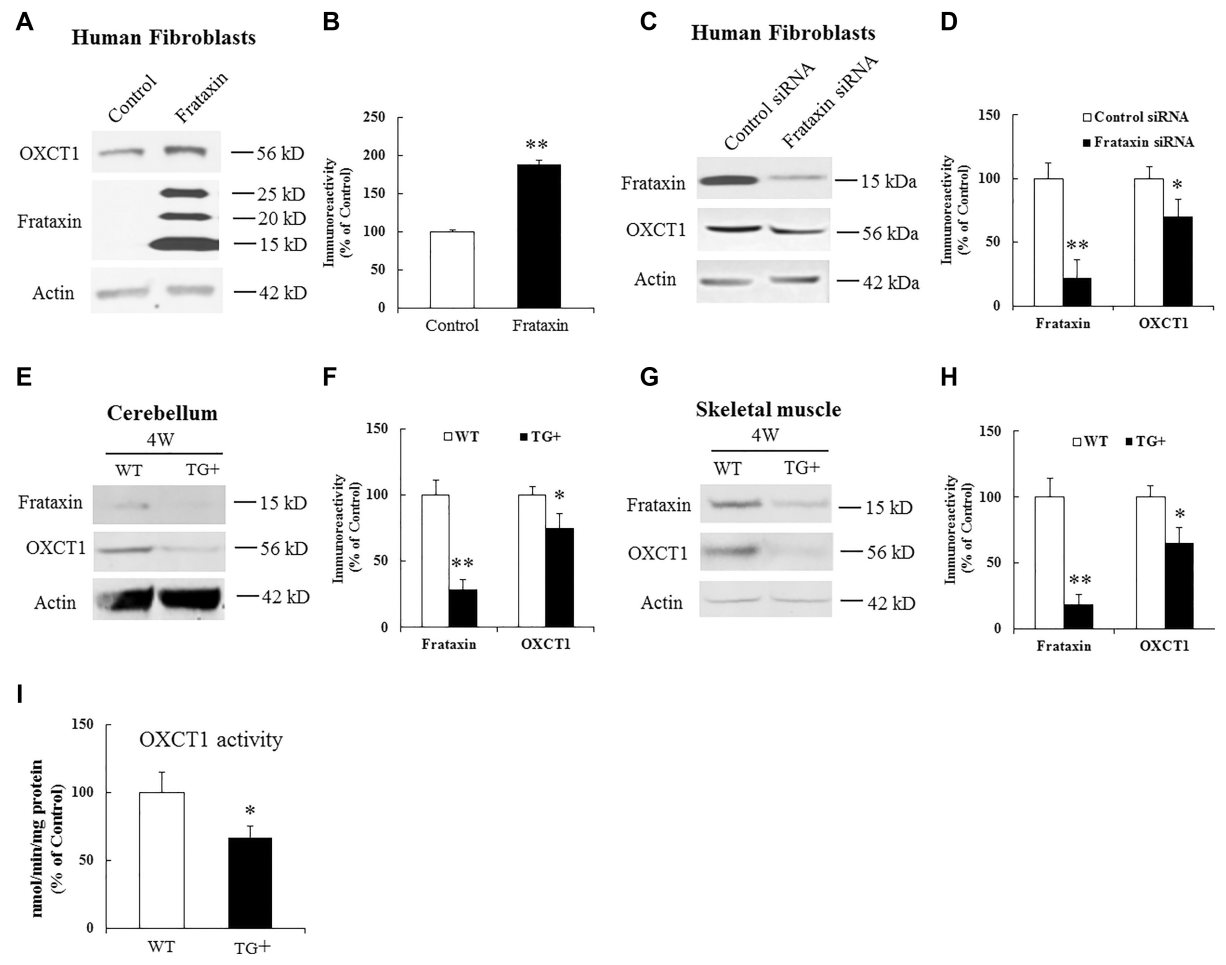
Effects of frataxin overexpression and knockdown on OXCT1 protein levels. Representative blots and bar graphs show increased OXCT1 in human skin fibroblasts transduced with lentivirus carrying frataxin gene for 5 days (A and B, *n* = 5) and decreased OXCT1 in human skin fibroblasts transfected with frataxin siRNA for 5 days (C and D, *n* = 5) as well as in cerebellum (E and F, *n* = 6) and skeletal muscle (G and H, *n* = 6) from frataxin knockdown mice induced with doxycycline for 4 weeks. Accordingly, OXCT1 activity was reduced in the cerebellum of frataxin knockdown mice (I). OXCT1 activity is shown as nmol of acetoacetyl-CoA produced per min per mg of total protein in cerebellar homogenates measured spectrophotometrically at 313 nm. *P < 0.05 and **P < 0.01. Data was expressed as mean ± SE (error bars).

The physical interaction between OXCT1 and frataxin prompted us to investigate the regulation of OXCT1 by frataxin. We first studied the effect of frataxin overexpression on OXCT1 levels. Transduction with lentivirus carrying pHAGE-*FXN* gene in human skin fibroblasts caused a significant increase in OXCT1 levels in comparison with vector control (Fig. 2A and B; 89% increase, *n* = 5, *P* < 0.05). Similar results were found in frataxin-transfected HEK293 cells (Figure S1A and S1B; 80% increase, *n* = 4, *P* < 0.01), suggesting that overexpression of frataxin increases OXCT1.

We next examined the effect of frataxin knockdown on OXCT1. Treatment with frataxin siRNA led to 22% residual frataxin levels compared with control (Fig. 2C and D; *n* = 5, *P* < 0.01), which was accompanied by a significant decrease in OXCT1 (Fig. 2C and D; 30% decrease, *n* = 5, *P* < 0.05). To examine whether frataxin deficiency decreases OXCT1 *in vivo* in affected tissues, both wildtype (WT) and doxycycline-inducible frataxin knockdown mice were treated with doxycycline for 4 weeks followed by Western blotting. In comparison with WT mice, doxycycline induction decreased frataxin levels in the homogenates of both cerebellum (Fig. 2E and F; 71% decrease, *n* = 6, *P* < 0.01) and skeletal muscle (Fig. 2G and H; 81% decrease, *n* = 6, *P* < 0.01) of frataxin knockdown mice. Frataxin knockdown also reduced OXCT1 in the homogenates of both cerebellum (Fig. 2E and F; 25% decrease, *n* = 6, *P* < 0.05) and skeletal muscle (Fig. 2G and H; 35% decrease, *n* = 6, *P* < 0.05). In cerebellum, OXCT1 reduction lasted through 18 weeks of doxycycline induction when frataxin was knocked down to 5% of control (Figure S2A and S2B; OXCT1: 71% reduction, *n* = 6, *P* < 0.05). These results suggest that frataxin deficiency decreases OXCT1 protein levels in multiple tissues both in an acute and subacute manner.

To confirm that OXCT1 catalytic activity also decreases with the fall in OXCT1 protein levels, cerebellum was harvested from WT or frataxin knockdown mice for OXCT1 activity assays. Compared with WT mice, OXCT1 catalytic activity was significantly decreased (Fig. 2I; 33% decrease, *n* = 9, *P* < 0.05) in the cerebellar homogenates of frataxin knockdown mice, suggesting that frataxin deficiency decreases OXCT1 activity as well.

### OXCT1 levels are decreased in the cerebellum and skeletal muscle of the frataxin deficient knock-in/knockout (KIKO) mouse model

**Figure 3.**
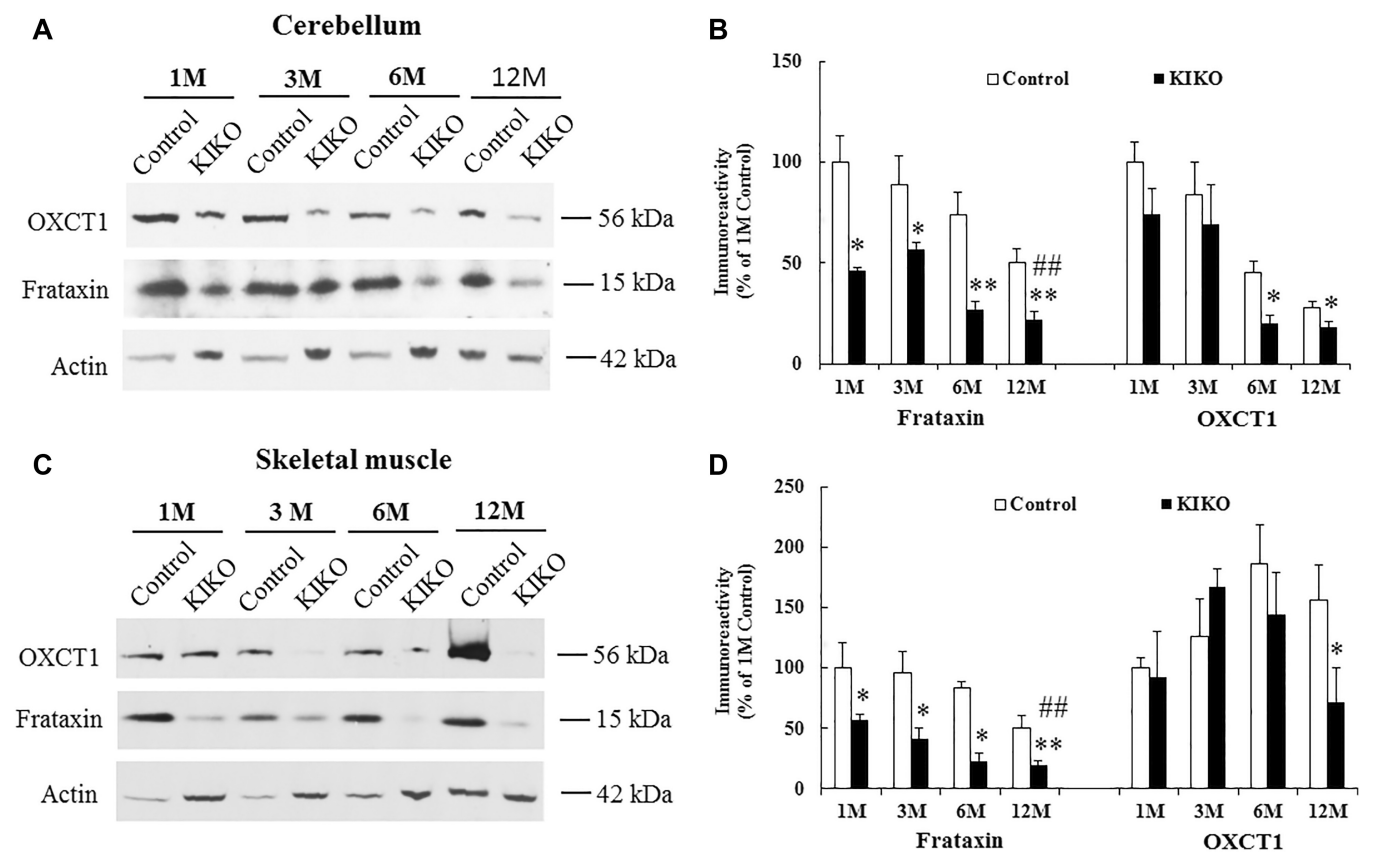
OXCT1 levels are decreased in both cerebellum and skeletal muscle of frataxin-deficient KIKO mice. Representative blots and bar graphs show decreased frataxin and OXCT1 levels in the homogenates of cerebellum (A and B, *n* = 4 to 7) and skeletal muscle (C and D, *n* = 4 to 7) of KIKO mice. Compared with 1M, frataxin levels of KIKO mice at 12M were also significantly decreased in both cerebellum (A and B) and skeletal muscle (C and D) (*n* = 4 to 7, ##*P* < 0.01). *P < 0.05 and **P < 0.01. Data was expressed as mean ± SE (error bars).

To examine whether chronic frataxin deficiency reduces OXCT1, we analyzed OXCT1 in the homogenates of both cerebellum and skeletal muscle from a frataxin-deficient KIKO mouse model at both asymptomatic (1, 3, and 6 months) and symptomatic ages (12 months and above). Frataxin levels of KIKO mice were significantly decreased in the homogenates of both cerebellum (Fig. 3A and B; 22% to 46% residual frataxin, *n* = 4 to 7,^*^P < 0.05, **P < 0.01) and skeletal muscle (20% to to 57% residual frataxin, n = 4 to 7, *P < 0.05, **P < 0.01) at all ages compared with controls. As shown previously (17), frataxin levels of KIKO mice also progressively decrease over time, with 22% and 20% residual frataxin levels observed at 12M in the homogenates of cerebellum (Fig. 3A and B; 1M: 46% residual frataxin, *n* = 4 to 7, ##*P* < 0.01) and skeletal muscle (Fig. 3C and D; 1M: 57% residual frataxin, *n* = 4 to 7, ##*P* < 0.01), respectively. Interestingly, OXCT1 levels of KIKO mice were also substantially reduced compared with control. OXCT1 reduction was observed at both 6M and 12M in cerebellar homogenates (Fig. 3A and B; 56% and 36% reduction for 6M and 12M, respectively, *n* = 4 to 7, *P* < 0.05) and at 12M in skeletal muscle homogenates (Fig. 3C and D; 65% reduction, *n* = 4 to 7, *P* < 0.05) of KIKO mice. As a negative control, the alpha subunit of pyruvate dehydrogenase (PDH), another mitochondrial protein involved in the influx of acetyl-coA from glycolysis into the TCA cycle, showed no change in the cerebellar homogenates of KIKO mice at either time point (Figure S3A and S3B). To rule out the possibility that OXCT1 reduction simply reflected decreased mitochondrial number in both the cellular and animal model of FRDA (17, 18), we analyzed mitochondrial copy number by measuring the ratio of mitochondrial DNA to nuclear DNA (mtDNA/nDNA) using quantitative PCR (qPCR) in the cerebellar homogenates of 12M KIKO mice. No change in mtDNA/nDNA (mt-ND1/Cftr) ratio was found in 12M KIKO mice compared with control mice (Figure S4A). We further measured mRNA levels of genes encoding mitochondrial proteins such as mt-CO1 (a mitochondrially encoded subunit of respiratory Complex IV) and COX7A (a nuclear-encoded subunit of respiratory Complex IV). No change in mRNA levels was detected for both genes in 12M KIKO mice compared with control mice (Figure S4B and S4C ), suggesting unaltered mitochondria copy number in 12M KIKO mice. These results suggest that OXCT1 reduction is not caused by decreased mitochondria copy number but a direct downstream event of chronic frataxin deficiency.

**Figure 4.**
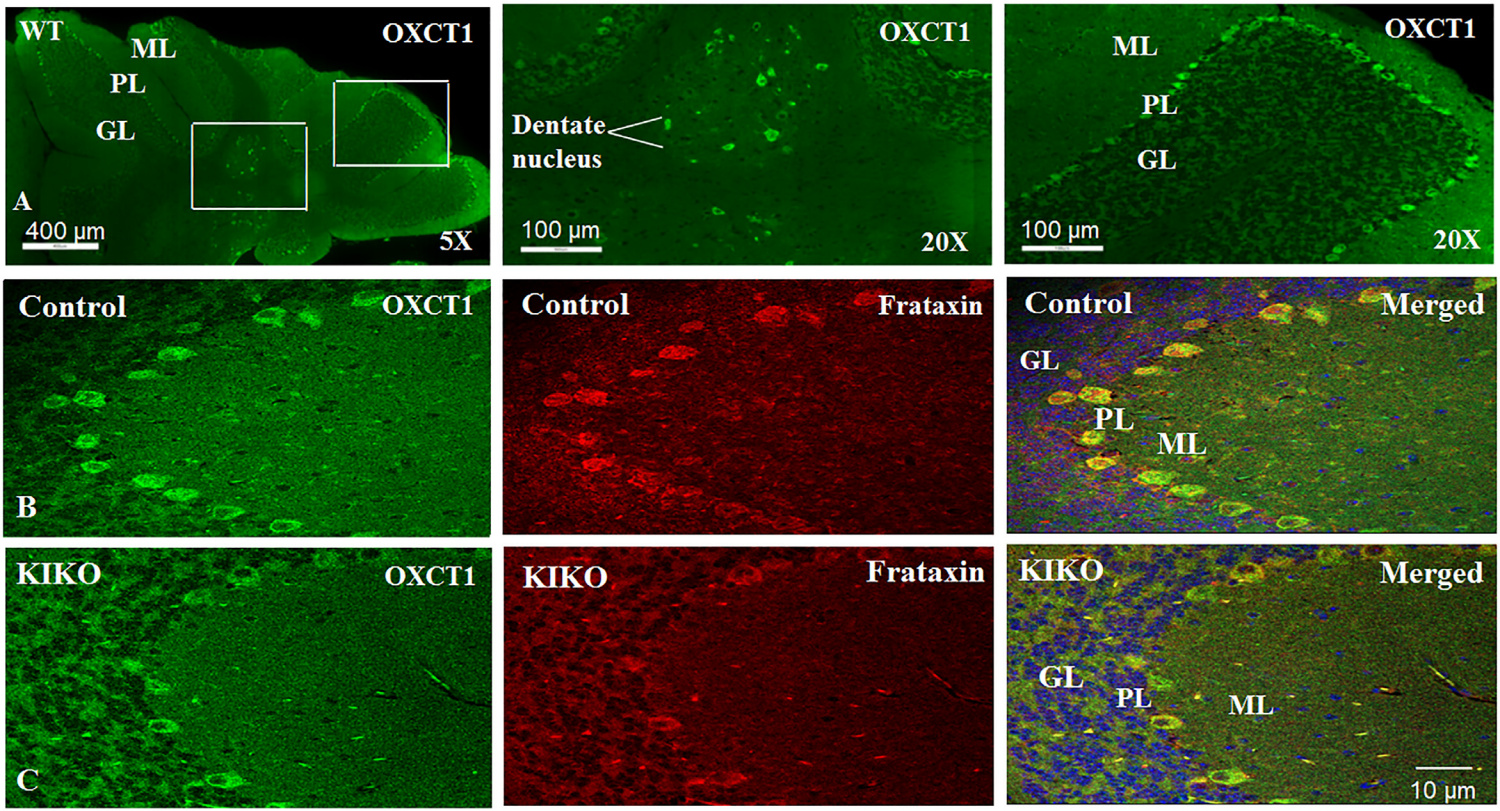
OXCT1 levels are reduced in the Purkinje neurons of the cerebellar cortex of KIKO mice. (A) Purkinje layer and dentate nucleus localization of OXCT1 in WT mouse cerebellum. Compared with control (B), both frataxin (red) and OXCT1 (green) immunoreactivity are lowered in the Purkinje neurons of KIKO mice (C). Merged images (B) and (C) showed the colocalization of frataxin and OXCT1 in the Purkinje neurons of mouse cerebellum. GL, granular layer; ML, molecular layer; and PL, Purkinje layer.

We next sought to investigate OXCT1 reduction in the intact cerebellum of symptomatic KIKO mice. Like frataxin (17), OXCT1 is predominantly located in the Purkinje layer and dentate nucleus (Fig. 4A). In KIKO mice, frataxin immunoreactivity was significantly decreased in the Purkinje neurons of cerebellar cortex (Fig. 4B and C). OXCT1 colocalized with frataxin in the Purkinje neurons (Pearson’s correlation coefficient: r = 0.638) and the levels of OXCT1 immunoreactivity were also significantly decreased compared with control, further confirming OXCT1 deficiency in KIKO mouse cerebellum. ATP5A levels (a subunit of respiratory Complex V) provided a negative control showing no change in immunoreactivity in frataxin-deficient Purkinje neurons (Figure S5A and S5B), consistent with the unaltered protein levels in the cerebellar homogenates of 12M KIKO mice in comparison with control mice (Figure S5C and 5D).

### OXCT1 levels are decreased in skeletal muscle of FRDA patients

**Figure 5.**
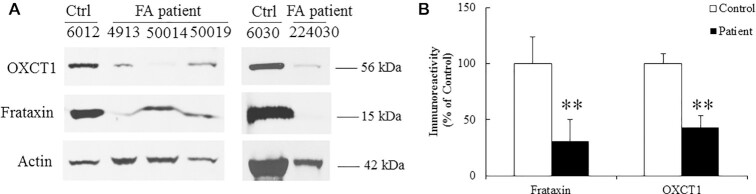
OXCT1 levels are decreased in the skeletal muscle of FRDA patients. Representative blots (A) and bar graph (B) demonstrate decreased OXCT1 and frataxin in the skeletal muscle of FRDA patients (*n* = 5, **P < 0.01). Data was expressed as mean ± SE (error bars).

To examine whether OXCT1 reduction occurs in the skeletal muscle of FRDA patients, skeletal muscle collected from FRDA patients was homogenized followed by western blotting. Compared with healthy individuals, both frataxin and OXCT1 levels were significantly decreased in the skeletal muscle homogenates of FRDA patients (Fig. 5A and B; 69% and 57% decrease for frataxin and OXCT1, respectively, *n* = 5,^**^P < 0.01), providing further evidence that frataxin deficiency decreases OXCT1 levels.

### Frataxin regulates OXCT1 through suppression of the ubiquitin–proteasome system dependent degradation

**Figure 6.**
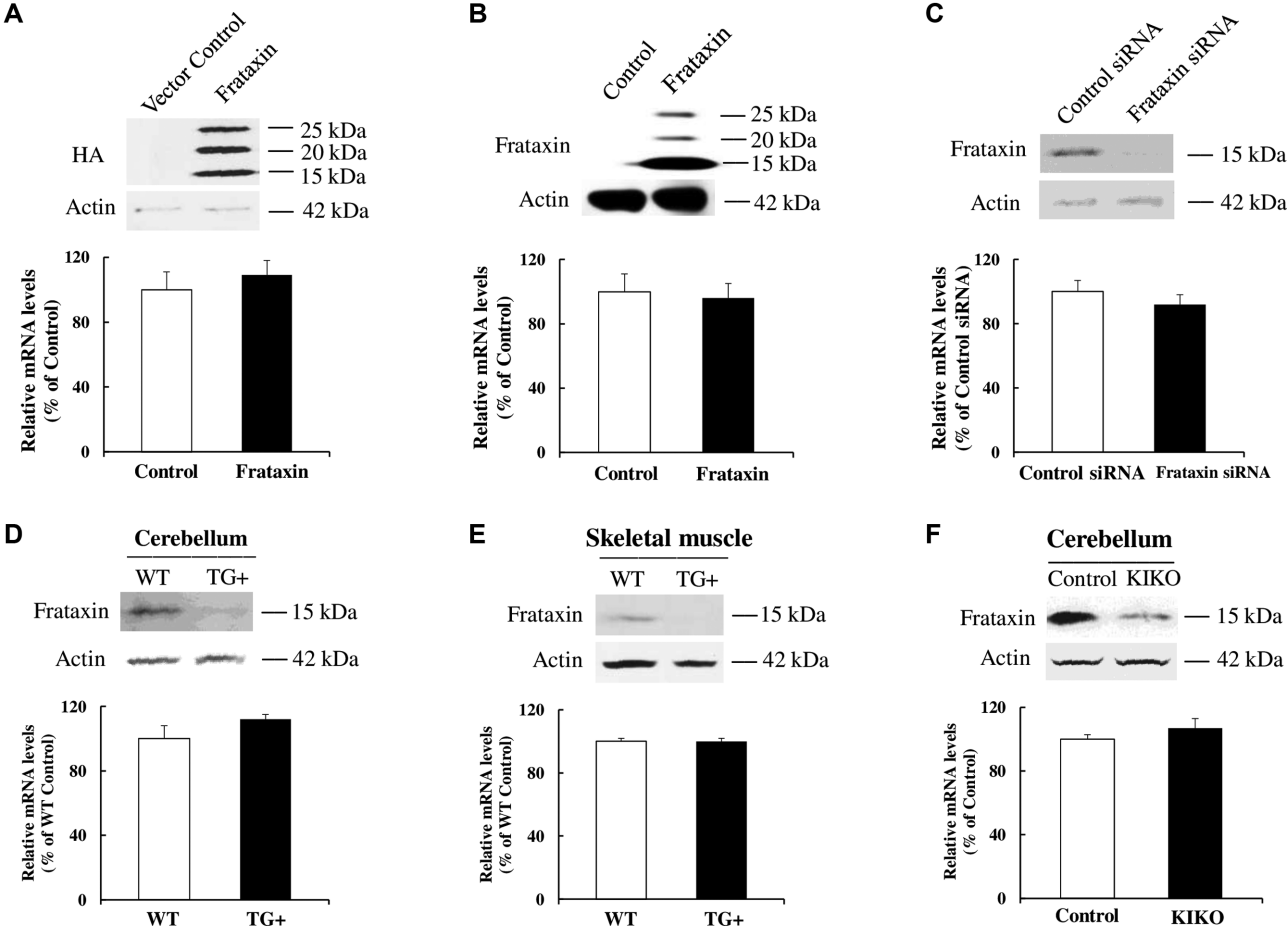
Frataxin overexpression or deficiency has no affect on OXCT1 mRNA levels. OXCT1 mRNA was quantified by RT-qPCR in: (A) HEK293 cells transfected with vector control or frataxin with a C-terminal HA tag for 24 h (*n* = 3), (B) human skin fibroblasts infected with lentivirus containing frataxin gene or vector control for 5 days (*n* = 3), (C) human skin fibroblasts transfected with control or frataxin siRNA for 5 days (*n* = 4), (D) cerebellum of frataxin knockdown mice induced with doxycycline for 4 weeks (*n* = 8), (E) skeletal muscle of frataxin knockdown mice induced with doxycycline for 4 weeks (*n* = 8), and (F) cerebellum of KIKO mice at 12M of age (*n* = 8). OXCT1 mRNA expression was normalized to actin.

To investigate the mechanism underlying the regulation of OXCT1 by frataxin, we examined OXCT1 mRNA levels in frataxin transfected HEK293 cells by reverse transcription quantitative polymerase chain reaction (RT-qPCR). While frataxin overexpression significantly increased OXCT1 protein levels (Figure S1A and S1B), no change in OXCT1 mRNA transcripts was detected compared with control cells (Fig. 6A; *n* = 3). Similarly, no change in OXCT1 mRNA transcripts was detected in frataxin transduced fibroblasts compared with control cells (Fig. 6B; *n* = 3). We next examined whether frataxin deficiency changes OXCT1 mRNA levels by RT-qPCR. Frataxin knockdown had no effect on OXCT1 mRNA levels either in fibroblasts (Fig. 6C; *n* = 4) or in tissues from frataxin knockdown mice treated with doxycycline for 4W (Fig. 6D and E; *n* = 8) or KIKO mice (Fig. 6F; *n* = 8), consistent with the result from human skin fibroblasts derived from FRDA patients (19). Taken together, these results suggest that the regulation of OXCT1 by frataxin is not mediated by changes in OXCT1 transcription.

**Figure 7.**
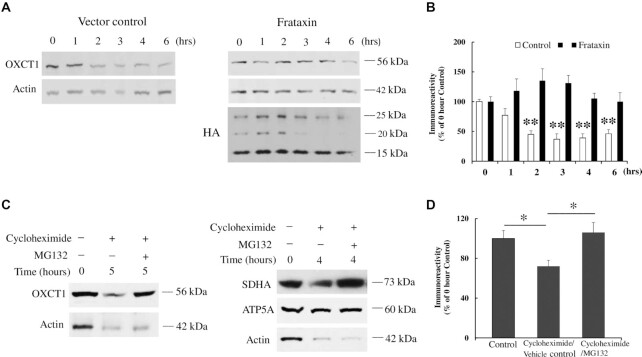
Frataxin regulates OXCT1 through suppression of UPS-dependent OXCT1 degradation. (A) Representative blots showing OXCT1 protein degradation over time in the presence of cycloheximide with or w/o frataxin overexpression in HEK293 cells. (B) Quantification of OXCT1 degradation in the presence of cycloheximide with or w/o frataxin overexpression in HEK293 cells. (C) Representative blots showing the blockade of OXCT1 degradation by MG132 (10 μM, 5 h) in HEK293 cells. SDHA and ATP5A were used as a positive and negative control of proteasome inhibition (10 μM MG132, 4 h), respectively. (D) Quantification of the blockade of OXCT1 degradation by MG132. *P < 0.05 and **P < 0.01. Data was expressed as mean ± SE (error bars).

We next investigated the effect of frataxin on OXCT1 protein turnover in the presence of cycloheximide, a protein synthesis inhibitor, in HEK293 cells transfected with vector control or frataxin with an HA tag added at the C-terminus. Cycloheximide treatment for 2 h decreased OXCT1 protein levels (Fig. 7A and B; 55% decrease, *n* = 4, **P < 0.01). This decrease continued until 6 h after cycloheximide treatment (Fig. 7A and B; 63%, 61%, and 64% decrease for 3, 4, and 6 h, respectively, *n* = 4, **P < 0.01). However, frataxin overexpression blocked the degradation of OXCT1 with no decrease detected at multiple time points (Fig. 7A and B; *n* = 4, *P* > 0.05). Seipin, a protein involved in energy homeostasis and lipid droplet biogenesis (20, 21) served as a negative control. Frataxin overexpression had no effect on seipin degradation in the presence of cycloheximide (Figure S6A and S6B; *n* = 4, *P < 0.05, #*P* < 0.05, **P < 0.01, and ##*P* < 0.01). We further investigated the proteolytic pathway involved in OXCT1 degradation. MG132, a proteasome inhibitor widely used in the ubiquitin–proteasome system (UPS) (22), blocked the degradation of OXCT1 in the presence of cycloheximide (Fig. 7C and D; *n* = 4, *P* < 0.05). SDHA and ATP5A served as positive and negative controls of proteasome inhibition, respectively (22) (Fig. 7C). These results demonstrate that frataxin regulates OXCT1 through inhibiting UPS-dependent degradation.

### Ketone body levels are increased in the plasma of frataxin-deficient KIKO mice after fasting

**Figure 8.**
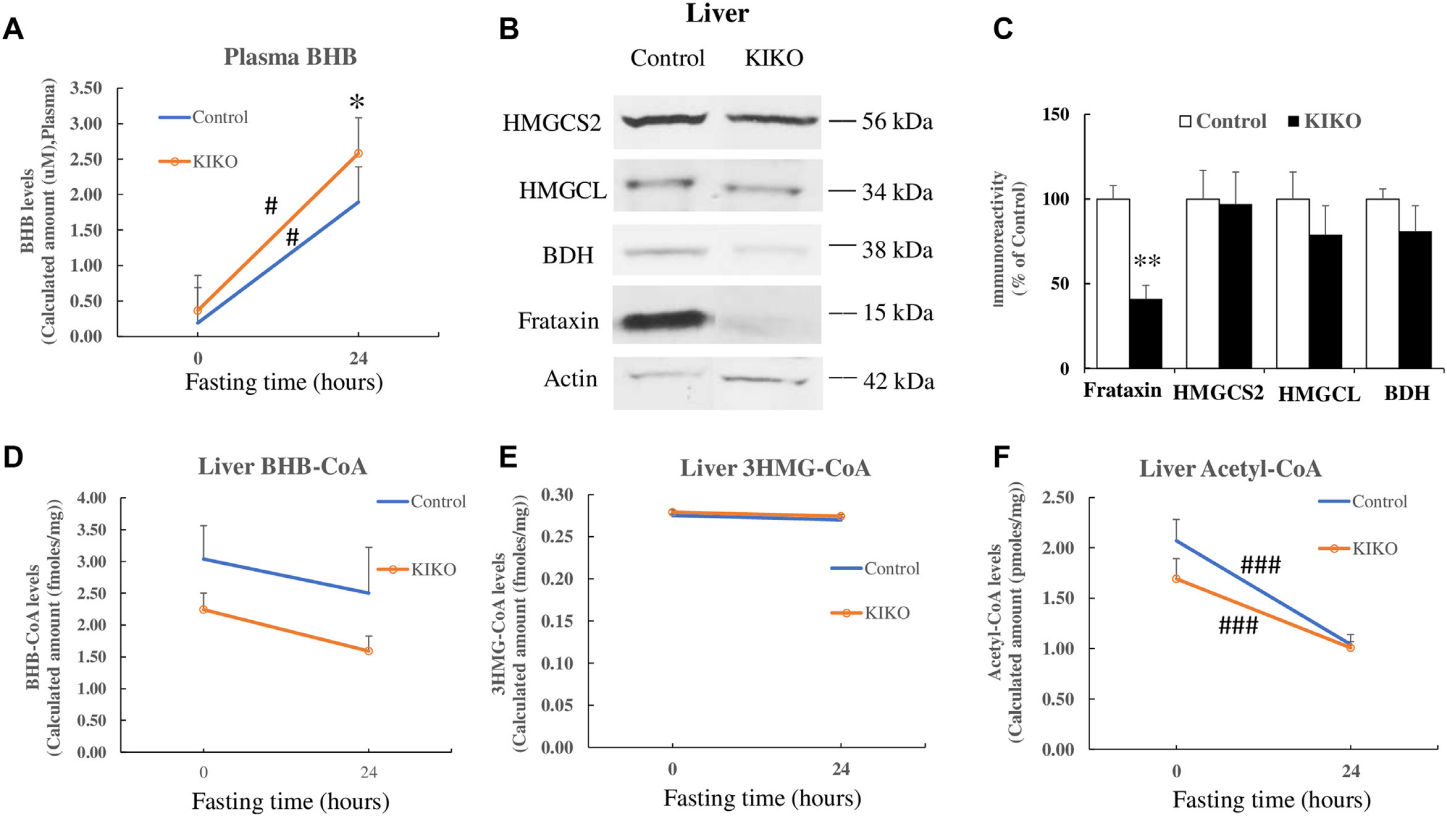
BHB levels are increased in the plasma of KIKO mice after fasting. (A) BHB levels in control and KIKO mice before and after fasting measured by mass spectrometry (*n* = 12 to 14). (B) and (C) Showing decreased frataxin levels in the liver of KIKO mice. No change in HMGCS2, HMGCL, and BDH levels was detected (*n* = 6). Similarly, no change in the levels of liver BHB-CoA (D) and 3HMG-CoA (E), measured by mass spectrometry, was detected while acetyl-CoA (F) was significantly decreased after fasting in both control and KIKO mice (*n* = 10 to 14). *P < 0.05, #*P* < 0.05, **P < 0.01, and ###P < 0.001. Data was expressed as mean ± SE (error bars).

As OXCT1 reduction decreases OXCT1 activity (Fig. 2I), we hypothesized that OXCT1 reduction in the cerebellum and skeletal muscle of KIKO mice would decrease ketone body utilization and increase ketone body levels in plasma. To confirm this hypothesis, KIKO mice at 18 to 26 M of age were fasted for 24 h followed by plasma collection and mass spectrometry analysis for ketone body levels. β-hydroxybutyrate (BHB) was measured because of its high stability compared with acetoacetate and acetone. Plasma BHB levels were significantly increased in both control (Fig. 8A; 8.87-fold increase over nonfasting condition, *n* = 13 for nonfasting and *n* = 12 for fasting condition, #*P* < 0.05) and KIKO mice (6.1-fold increase over nonfasting condition, *n* = 14 for both nonfasting and fasting condition, #*P* < 0.05) after fasting. In comparison with controls, plasma BHB levels were also significantly elevated in KIKO mice after fasting (Fig. 8A; 36% increase, *n* = 12 for control and *n* = 14 for KIKO mice,^*^P < 0.05), suggesting a compromised utilization or increased production of ketone body in KIKO mice. To rule out the possibility that BHB elevation is caused by increased ketone body production, we measured the levels of 3-hydroxy-3-methylglutaryl-CoA synthase 2 (HMGCS2), 3-hydroxy-3-methylglutaryl-CoA lyase (HMGCL), and beta-Hydroxybutyrate dehydrogenase (BDH), enzymes involved in ketone body production, in the liver of KIKO mice at 18M of age by western blotting. While frataxin levels were significantly reduced in the liver of KIKO mice (Fig. 8B and C; 59% decrease, *n* = 6, *P < 0.05), no change in HMGCS2, HMGCL, or BDH levels was detected (Fig. 8B and C; *n* = 6, *P* > 0.05). In support of these data, no changes in metabolic intermediates of ketone body production in liver such as BHB-CoA and 3HMG-CoA were detected (Fig. 8D and E; *n* = 10 to 14 for both nonfasting vs. fasting condition and control vs. KIKO mice, *P* > 0.05) while Acetyl-CoA levels were significantly decreased after fasting in both control and KIKO mice (Fig. 8F; *n* = 10 to 14 for both nonfasting vs. fasting condition and control vs. KIKO mice, ###*P* < 0.001), reflecting a rapid decrease in glycolysis. These results suggest that ketone body utilization defect but not increased production contribute to plasma BHB elevation in KIKO mice.

As ketone body utilization involves the conversion of ketone body to acetoacetyl-CoA by OXCT1 and the cleavage of acetoacetyl-CoA into two acetyl-CoA by the enzyme thiolase, we next examined thiolase in the cerebellar homogenates of KIKO mice. While OXCT1 was significantly reduced in the cerebellar homogenates of KIKO mice (Fig. 3A and B), no change in thiolase levels was found in comparison with control (Figure S7A and S7B; *n* = 6 to 8), suggesting that OXCT1 reduction is the major factor leading to ketone body utilization deficit and elevated plasma BHB.

### Acetyl-CoA levels are increased in skeletal muscle of control but not frataxin-deficient KIKO mice after fasting

**Figure 9.**
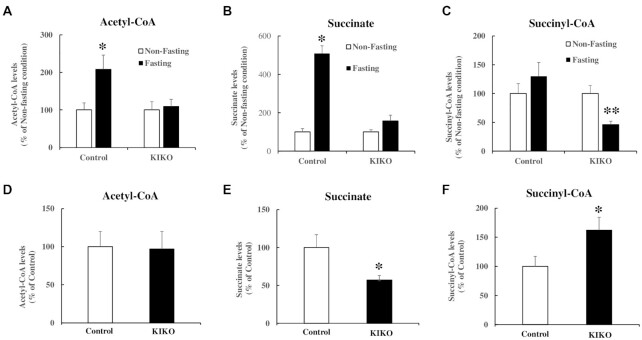
Acetyl-CoA levels are increased in skeletal muscle homogenates of control but not KIKO mice after fasting. Mass spectrometry was performed to measure acetyl-CoA, succinate, and succinyl-CoA in skeletal muscle of control and KIKO mice in both nonfasting and fasting conditions. Fasting increased acetyl-CoA (A) and succinate (B) in control but not KIKO mice compared with nonfasting condition (*n* = 10 to 12 for control mice and *n* = 13 for KIKO mice for both fasting and nonfasting condition). Succinyl-CoA stayed unchanged in control but significantly decreased in KIKO mice after fasting (C) (*n* = 10 to 12 for control mice and *n* = 13 for KIKO mice for both fasting and nonfasting condition). Under nonfasting condition, no change in acetyl-CoA was found between control and KIKO mice (D) while a significant decrease in succinate (E) and a significant increase in succinyl-CoA levels (F) were detected in KIKO mice. *P < 0.05 and **P < 0.01. Data was expressed as mean ± SE (error bars).

As plasma ketone body levels were significantly increased in frataxin-deficient KIKO mice compared with control mice (Fig. 8A), we hypothesized that plasma ketone body elevation would accompany decreased acetyl-CoA in tissues of KIKO mice. Mass spectrometry analysis was performed in the skeletal muscle homogenates of control and KIKO mice under both nonfasting and fasting conditions. While no change was detected in acetyl-CoA between control and KIKO mice under nonfasting conditions (Fig. 9D), fasting significantly increased acetyl-CoA in the skeletal muscle homogenates of control (108% increase, *n* = 10 to 12,^*^P < 0.05) but not KIKO mice (*n* = 13, *P* > 0.05) compared with nonfasting condition (Fig. 9A). Accordingly, succinate, the metabolite of succinyl-CoA, was significantly increased (Fig. 9B; 409% increase, *n* = 10 to 12, *P < 0.05) while succinyl-CoA remained unchanged in control mice (Fig. 9C; *n* = 10 to 12, *P* > 0.05) after fasting. In KIKO mice, no change in succinate levels was detected (Fig. 9B; *n* = 13, *P* > 0.05) while succinyl-CoA levels were significantly decreased after fasting (Fig. 9C; 54% decrease, *n* = 13, **P < 0.01). We also noticed a significant decrease in succinate (43% decrease, *n* = 13, *P < 0.05; Fig. 9E) and a significant increase in succinyl-CoA levels (62% increase, *n* = 13, *P < 0.05; Fig. 9F) between control and KIKO mice under nonfasting condition, suggesting a possible anaplerotic effect in frataxin-deficient cells. Taken together, these results suggest that ketone body utilization deficit occurs in skeletal muscle of KIKO mice.

### BHB increases acetyl-CoA in control but not frataxin deficient C2C12 cells

**Figure 10.**
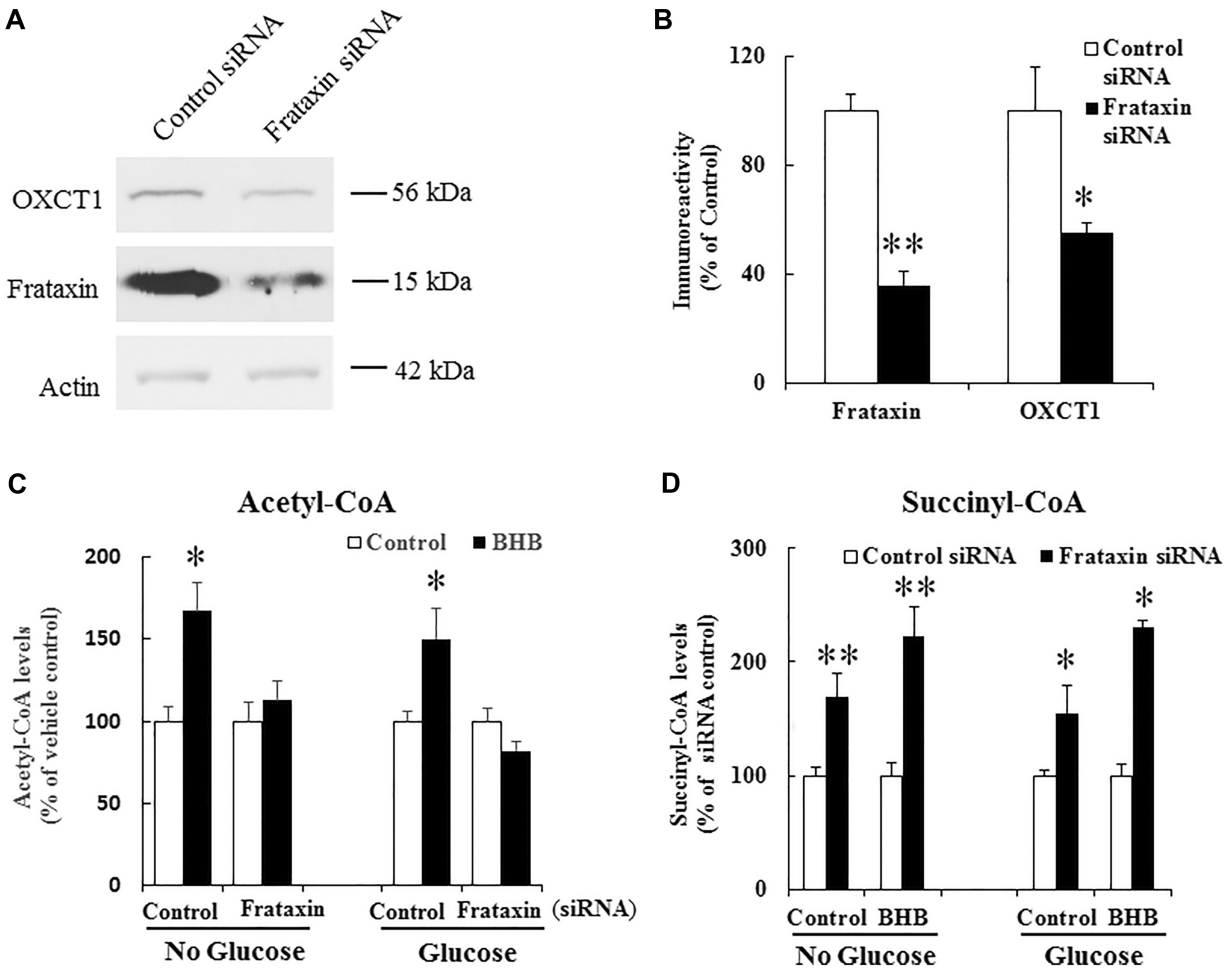
BHB increases acetyl-CoA in control but not frataxin deficient C2C12 cells. (A) Representative blots showing decreased frataxin and OXCT1 levels in differentiated C2C12 cells treated with control or frataxin siRNA. (B) Quantification of frataxin and OXCT1 levels (*n* = 7 to 9). (C) 10 mM BHB treatment for 24 h increased acetyl-CoA levels, measured by mass spectrometry, in control but not frataxin siRNA treated C2C12 cells both in medium with or without glucose (*n* = 5 to 6 for no glucose condition and *n* = 4 to 5 for glucose condition). (D) Succinyl-CoA levels were increased in frataxin siRNA treated C2C12 cells treated with vehicle control or BHB both in medium with or without glucose (*n* = 5 to 6 for no glucose condition and *n* = 4 for glucose condition). *P < 0.05 and **P < 0.01. Data was expressed as mean ± SE (error bars).

To confirm the role of OXCT1 reduction in ketone body utilization at cellular level, differentiated C2C12 cells, a mouse skeletal muscle cell line, were subjected to frataxin knockdown followed by BHB treatment for 24 h in medium with or without glucose. Cellular acetyl-CoA contents were measured by mass spectrometry analysis. As shown in Fig. 10A and 10B, treatment with frataxin siRNA led to 36% residual frataxin (*n* = 9, **P < 0.01) accompanied by a significant decrease in OXCT1 (45% decrease, *n* = 7, *P < 0.05). While BHB treatment significantly increased acetyl-CoA contents in scrambled siRNA treated control cells cultured in medium with or without glucose (Fig. 10C; no glucose: 67% increase, *n* = 5 to 7, *P < 0.05; glucose: 50% increase, *n* = 5 to 6, *P < 0.05), no change in acetyl-CoA was found in frataxin knockdown cells at either condition (*n* = 5 to 7 for no glucose condition and *n* = 5 to 6 for glucose condition). Accordingly, succinyl-CoA contents were significantly increased in frataxin knockdown cells treated with BHB both in medium with or without glucose (Fig. 10D; no glucose: 122% increase from siRNA control, *n* = 5 to 6, **P < 0.01; glucose: 130% increase from siRNA control, *n* = 4, *P < 0.05). Although vehicle control-treated frataxin knockdown cells also have an increase in succinyl-CoA levels both in medium with or without glucose (no glucose: 68% increase from siRNA control, *n* = 5 to 6, **P < 0.01; glucose: 55% increase from siRNA control, *n* = 4, *P < 0.05), the degree of increase is much smaller compared with BHB treated frataxin knockdown cells, suggesting a specific effect of OXCT1 in ketone bodies utilization as well as potentially an allosteric or anaplerotic increase in succinyl-CoA levels in frataxin depleted cells. These results demonstrate that OXCT1 reduction leads to ketone body utilization deficits*in vitro* in frataxin deficient cells as well.

### Clinical relevance of OXCT1 deficiency

**Figure 11.**
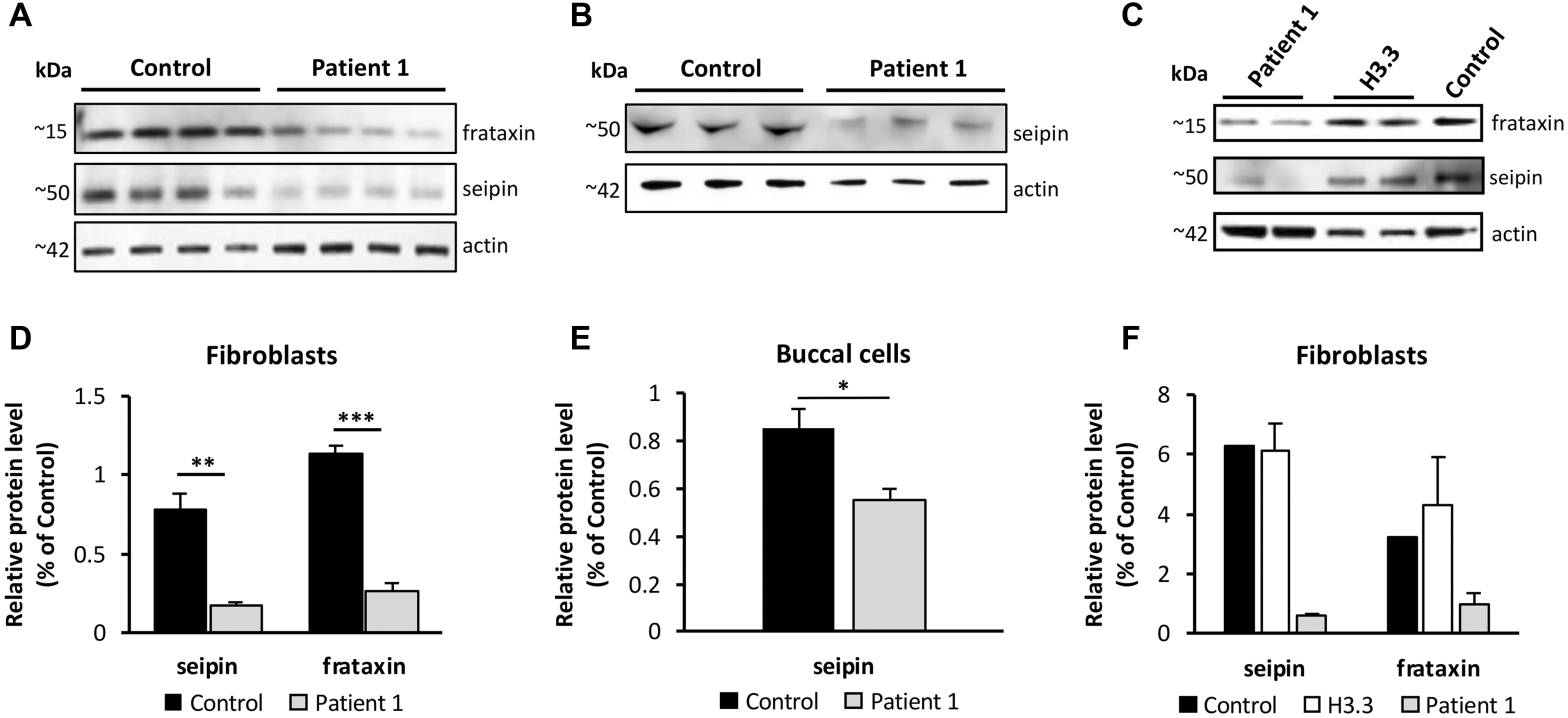
Decreased seipin and frataxin levels in patient 1 fibroblasts and buccal cells. (A) and (B) Western blot analysis of seipin and frataxin levels in fibroblasts and buccal cells from patient 1. (C) Western blot analysis of seipin and frataxin levels in fibroblasts from H3.3 patient. (D) and (E) Quantification of seipin and frataxin levels in fibroblasts and buccal cells from patient 1. (F) Quantification of seipin and frataxin levels in fibroblasts from H3.3 patient. *N* = 14 technical repeats.^*^P < 0.05, **P < 0.01, and ***P < 0.01. Data was expressed as mean ± SE (error bars).

While ketosis (outside of diabetic ketoacidosis) is not a known feature of FRDA, we reasoned that the relative deficiency of OXCT1 might elevate blood ketones in selected situations. We identified two siblings with FRDA who exhibit recurrent ketosis when exposed to a high-fat diet (Supplementary text), prompting the search for a second disorder via research-based whole-exome sequencing. Homozygous variants (p.A282T) identified in *BSCL2* (which codes for seipin) found in both patients suggest that relative deficiency of seipin, which leads to dysregulated fatty acid breakdown and dysfunction in the initial steps in lipid droplet synthesis (23, 24), could contribute to their overall phenotype in combination with other aspects of FRDA. Fibroblasts from patient 1 had an 80% decrease in seipin levels compared to control while buccal cells had a 30% decrease in such levels. Frataxin protein levels decreased 80% in comparison to control fibroblasts (Fig. 11A, B, D, and E). In addition, seipin and frataxin levels did not significantly change in fibroblasts from a patient bearing de novo germline mutations in histone 3 family 3B (H3.3) used here as a control for an unrelated disease (Fig. 11C and F). These identify the *BSCL2* variants as mutants that lead to relative seipin deficiency independent of their FRDA, which should rapidly increase fatty acid breakdown. As systemic ketosis is not found in other patients with seipin deficiency (25–28), this identifies the biochemical abnormalities in FRDA, in particular the potential importance of OXCT1 deficiency (Figure S8), as crucial for the severe ketosis in these two patients in FRDA.

## Discussion

In the present study, we demonstrate that frataxin controls ketone body metabolism through regulation of the UPS-dependent turnover of OXCT1. Frataxin physically interacts with OXCT1 to control levels of OXCT1 as evidenced by up and down-regulated levels of OXCT1 with frataxin overexpression and deficiency, respectively. Frataxin deficiency leads to OXCT1 reduction in multiple cell types along with decreased activity and compromised ketone body metabolism both *in vivo* and *in vitro*. These results demonstrate a direct role of frataxin in ketone body metabolism and identify OXCT1 as a mediator of frataxin deficiency-induced changes in FRDA. Such results, to our knowledge, have not been noted in other mitochondrial diseases nor in models of global mitochondrial dysfunction.

Germline OXCT1 KO mice exhibit no ketone body oxidation in any tissue and die within 2 days after birth due to hyperketonemic hypoglycemia (13), demonstrating the key role of OXCT1 in tissue utilization of ketone body. Both brain and skeletal muscle are among the tissues utilizing high amounts of ketone bodies. We found a substantial reduction of OXCT1 in the cerebellum (56% and 36% reduction at 6 M and 12 M, respectively) and skeletal muscle (54% reduction) of KIKO mice as well as in the skeletal muscle of FRDA patients. In Purkinje neurons, OXCT1 reduction correlates with frataxin deficiency. In parallel, plasma ketone body levels are significantly elevated in KIKO mice. This elevation is not caused by increased ketone body production as no change in the enzymes in the ketogenesis pathway such as HMGCS2, HMGCL, and BDH was found, supported by the lack of changes in the levels of intermediate metabolites such as BHB-CoA and 3HMG-CoA. In examining ketone body utilization pathway, we found no change in thiolase levels in the cerebellar homogenates of KIKO mice. Functionally, succinyl-CoA levels were significantly increased while acetyl-CoA were significantly decreased *in vitro* in frataxin deficient and OXCT1 reduced C2C12 cells treated with a ketone body. In agreement with *in vitro* data, acetyl-CoA was significantly increased in skeletal muscle homogenates of control but not frataxin-deficient and OXCT1-reduced KIKO mice. These results demonstrate that OXCT1 reduction triggers tissue utilization deficits and compromised ketone body metabolism. OXCT1 deficiency also explains the ketosis seen in FRDA patients carrying *BSCL2* variants when exposed to a high fat diet, and emphasizes the features of FRDA as a specific metabolic disorder in addition to being a disorder of mitochondrial function.

As prolonged exercise stimulates the capacity of skeletal muscle to take up ketones from blood (29), OXCT1 reduction in the skeletal muscle of FRDA patients could contribute to prolonged postexercise recovery and exercise intolerance (11, 12, 30, 31), a significant clinical feature of early FRDA. Exercise intolerance in KIKO mice (at as early as 6 months of age) occurs without a loss of muscle mass or strength (32), suggesting a metabolic deficit. As exercise training increases OXCT1 activity (up to a 2-fold change) and ketone body oxidation in the skeletal muscle of rats (33, 34), changes in OXCT1 could explain the observation that long term endurance exercise training prevents the onset of exercise intolerance in KIKO mice without restoring frataxin function (32). Our results provide a molecular mechanism that could explain the exercise intolerance in patients of FRDA and also point to the importance of exercise for FRDA patients in maintaining abilities.

The present results may also explain other components of the features of FRDA. ATP deficiency appears to be a crucial aspect of the disease both *in vitro* and *in vivo* (12, 30, 31). In conjunction with deficiency of active mitochondrial aconitase and dysfunction in glycolysis, the inability to utilize ketone bodies would limit the ability of frataxin-deficient cells to produce ATP, as evidenced by the failure of ketone body to be metabolized to acetyl-CoA in C2C12 cells and skeletal muscle of KIKO mice. This would be most prominent in times of stress, including not only exercise but also during fasting (as occurs perioperatively) and conceivably other times. This could explain not only unexpected issues in prolonged scoliosis surgery noted in FRDA but also the baseline progression of the disease. Interestingly, frataxin deficiency alone does not explain the unique anatomical progression of FRDA, as almost all cells have similar levels of frataxin in FRDA patients (1, 4). Instead, the major affected tissues in FRDA (brain, heart, and to a lesser degree skeletal muscle) match the tissue distribution of OXCT1. Further studies examining the detailed anatomical distribution of OXCT1 in the CNS may better test the meaning of this correlation. Still, the data here suggest that the tissue-selective pathology of FRDA may reflect OXCT1 distribution to a significant degree.

Ketone bodies have strong antioxidant capacity via directly scavenging free radicals (35) and indirectly inhibiting mitochondrial production of reactive oxygen species through increasing the [NAD+]/[NADH] ratio (36). Deficiency of ketone body metabolism increases oxidative stress. Hearts from mice with cardiomyocyte-specific loss of OXCT1 exhibits signatures of oxidative stress, including superoxide accumulation and myocardial protein carbonylation, along with structural and hemodynamic abnormalities (37). In addition, exogenous administration of ketone bodies protects neonatal neurons and myocardium against oxidative stress leading to increases in neuronal viability and enhanced contractile performance, respectively (38, 39). While ketone body metabolism deficiency causes oxidative stress, the latter also decreases OXCT1 activity (40), potentially creating a toxic feedback. Oxidative stress has been found in many cellular and animal models of FRDA as well as patient cells (41–44). Skeletal muscle of KIKO mice also undergoes oxidative stress as evidenced by increased 4-hydroxynonenal, which is ameliorated by exercise training (32). OXCT1 reduction thus might be one factor contributing to disease progression in FRDA.

The mechanistic study on the regulation of frataxin on OXCT1 demonstrates that frataxin inhibits UPS-dependent degradation of OXCT1, but has no effect on the transcription of the *OXCT1* gene as demonstrated by unaltered OXCT1 mRNA levels in multiple cell types and tissues under both frataxin overexpression and deficiency conditions. This effect is specific to OXCT1 as frataxin has no effect on the degradation of seipin, suggesting that the binding of frataxin to OXCT1 directly impedes OXCT1 turnover. This explains the rapid course of OXCT1 decreases following siRNA knockdown and the increases in OXCT1 with overexpression of frataxin in normal cells. Our results also agree with the concept that mitochondrial proteins are subject to UPS-dependent regulation (22, 45). The precise role of frataxin in the UPS proteolytic pathway remains to be clarified, although a simple explanation is that the binding of frataxin sterically hinders ubiquitination of OXCT1. Further study will be needed to investigate the molecular interaction among frataxin, OXCT1, and the UPS pathway. Understanding such events may provide further understanding of the abnormal metabolic events in FRDA.

In conclusion, we have demonstrated a new role of frataxin in energy metabolism by regulating the turnover of OXCT1. Monitoring OXCT1 activity through genetic manipulation or posttranslational modification such as UPS might produce a beneficial effect on the quality of life for FRDA patients.

## Materials and Methods

### Animals

C57BL/6 mice (stock no: 000664) and frataxin knock-in/knockout (KIKO) mice (B6.Cg-Fxn^tm1.1Pand^ Fxn^tm1Mkn^/J; stock no. 012329) were purchased from Jackson Laboratory. Frataxin knockdown transgenic (TG) mice were obtained from Drs. Geschwind and Chandran at University of California Los Angeles, then bred with C57BL/6 mice to generate WT and TG mice. Mice handling and treatment were in accordance with standard regulations approved by the Children’s Hospital of Philadelphia Institutional Animal Care and Use Committee (IACUC; protocol 16–250). For the induction of frataxin knockdown, 2∼6-months-old mice were fed with chow containing 200 PPM doxycycline (Animal Specialties and Provisions, LLC., Quakertown, PA) or regular diet for 4 or 18 weeks before euthanasia.

### Preparation of tissue homogenates

After mice were euthanized, the cortex, cerebellum, skeletal muscle, and liver were collected and homogenized as described previously (46). The homogenates were spun down at 13,000 rpm for 15 min and the supernatant was stored at −80°C until use.

### Cell cultures

Primary rat cortical neurons from Sprague Dawley rat embryos at day 17 were isolated and cultured as described previously (46). The mouse myogenic C2C12 cells were cultured in Dulbecco’s modified Eagle’s medium (DMEM) with 10% fetal bovine serum, 2% glutamine, and 1% penicillin–streptomycin at 37 °C and 5% CO_2_. Once cells reach 70% confluency, they were differentiated with medium containing DMEM, 2% glutamine, 2% horse serum, and 1% penicillin–streptomycin for at least 5 days. Medium was changed every other day. Frataxin knockdown was carried out by the method below. Cells were then treated with 10 mM (R)-(−)-3-hydroxybutyric acid sodium salt (BHB; Santa Cruz biotech, Dallas, TX) in medium with or without glucose for 24 h and harvested for mass spectrometry analysis.

Patient 1, control, and H3.3 primary skin fibroblasts were provided by Metabolism Core and Dr. Elizabeth Bhoj at the Children’s Hospital of Philadelphia, respectively using an IRB‐approved protocol. The fibroblasts were cultured as previously described (46).

### RNA interference-mediated down-regulation of frataxin and lentiviral transduction of frataxin in human skin fibroblasts

Frataxin knockdown and overexpression were described previously (46). Briefly, cultured skin fibroblasts were transfected with human frataxin siRNA (Origene Technologies, Rockville, MD) or scrambled siRNA using Lipofectamine RNAiMax reagent (Thermo Fisher Scientific Inc., Waltham, MA) for 5 days. Cells were then lysed by Laemmli sample buffer (50 m m Tris–HCl, pH 6.8, 2% SDS, 5 m m EDTA, 0.1% bromophenol blue, 10% glycerol, and 2% β-mercaptoethanol) for western blot analysis. For frataxin overexpression in skin fibroblasts, lentivirus containing the frataxin gene (Genscript, Piscataway, NJ) or vector control (pHAGE-CMV-dsRed-UBC-GFP-W; Addgene, Watertown, MA), was transduced into fibroblasts with polybrene (8 μg/ml; Sigma, St Louis, MO) for 1 day. Medium was then changed to normal culture medium. Fibroblasts were collected after 5 days transduction.

### Co-immunoprecipitation

Mouse brain homogenates or mouse cortical neuronal lysates prepared as described previously (46) were immunoprecipitated with antibody against frataxin (Abcam, Cambridge, MA) overnight at 4°C. On the second day, protein G beads were added and incubated with the lysates for 2 h at 4°C. Beads were then spun down at 500 *g* for 5 min and washed four times with RIPA buffer. A volume of 50 μl of Laemmli sample buffer was then added and the samples were boiled for 5 min before SDS-PAGE.

### Western blot

Western blot was performed as described previously (46). The following antibodies were used: OXCT1 (Thermo Fisher Scientific Inc., 1/1000), frataxin (Abcam, 1/500), ISCU2 (Proteintech, Rosemont, IL, 1/1000), pan-actin (Cell signaling, Danvers, MA, 1/1000), SDHA (Cell signaling, 1/1000), ATP5A (Abcam, 1/1000), HMGCS2 (Cell signaling, 1/1000), HMGCL (GeneTex, Irvine, CA, 1/1000), BDH (Proteintech, 1/1000), seipin (Abnova, Taipei, Taiwan, 1/2000), and thiolase (Sigma, 1/1000).

### Protein stability assay

HEK293 cells were cultured as previously described (46). Cells were transfected with cDNAs for vector control or WT frataxin with a C-terminal HA tag for 24 h and then treated with cycloheximide (50 μg/ml, Sigma) or vehicle control for different amount of time (0, 1, 2, 3, 4, and 6 h). For MG132 experiment, cells were treated with cycloheximide along with MG132 (10 μM, Pubchem, Bethesda, MD) or vehicle control for 5 h for OXCT1 or 4 h for SDHA and ATP5A. After treatment, cells were lysed for western blot analysis.

### Total DNA and RNA isolation and quantification

Total DNA was extracted from the cerebellum of control and KIKO mice at 12 M of age using DNeasy blood and tissue kit (Qiagen, Valencia, CA). As previously described (47), total RNA was extracted from HEK293 cells transfected with plasmids containing WT frataxin with a C-terminal HA tag or vector control, human skin fibroblasts transduced with lentivirus containing the frataxin gene or vector control, human skin fibroblasts transfected with control or frataxin siRNA, cerebellum and skeletal muscle from control and frataxin knockdown mice treated with doxycycline for 4 weeks, and cerebellum from control and KIKO mice at 12 M of age. Both DNA and RNA were quantified by a NanoDrop 2000 Spectrophotometer (Thermo Scientific, Waltham, MA).

### Quantitative polymerase chain reaction (qPCR)

iScript cDNA synthesis kit (Bio-Rad, Hercules, CA) was used for cDNA synthesis from mRNA. qPCR was performed using SYBR kit (Qiagen, Hilden, Germany) in Applied Biosystem Real Time PCR instrument (Thermo Fisher Scientific). The primer sequences for mt-ND1, Cftr, mt-CO1, COX7a, human OXCT1, and actin were used as previously described (18, 48–51). Mouse OXCT1 primers were: forward: 5′-GGGCCCATACCCACTGAAAGA-3′; reverse: 5′-GACATGTCCCCCTCTAATCATGG-3′. Both primers were synthesized by Integrated DNA Technologies, Inc. (Skokie, IL). mt-CO1, COX7a, and OXCT1 gene transcripts were normalized to the expression levels of beta actin.

### Immunofluorescence

Immunofluorescence was performed as previously described (46). The following antibodies were used: OXCT1 (Thermo Fisher Scientific Inc., 1/200) and frataxin (Neuromab, Davis, CA, 1/100).

Immunohistochemical studies were performed as previously described (17). Briefly, KIKO mice and knock-in/wildtype (KIWT) controls of same gender at 12 months of age were perfused with 4% paraformaldehyde. The cerebella were harvested and fixed in 4% paraformaldehyde overnight followed by embedding in paraffin and sectioning (5 μM each; The Pathology Core at the Children’s Hospital of Philadelphia). The sections were blocked with 5% normal goat serum made with 0.3% (v/v) Triton X-100 in PBS at room temperature for 1 h followed by primary antibodies incubation at 4°C overnight. ATP5A was from Abcam (1/100). Second antibodies were then added and images were taken by Leica TCS SP8 confocal laser scanning microscope.

### OXCT1 activity assay

OXCT1 catalytic activity was measured as previously described (52, 53). Briefly, mouse cerebellar tissue was homogenized in TBST (12.5 mM Tris–HCl (pH 7.5), 75 mM NaCl, 25 mM KCl, and 0.05% Tween‐20) with protease inhibitors (Roche, 1/500). The homogenate was spun down at 20,000 *g* at 4°C for 20 min and the supernatants were used for activity assay. Assay contained 100 μg protein, 50 mM Tris–HCl, pH 8.5, 0.2 mM succinyl-CoA, 5 mM lithium acetoacetate, 5 mM MgCl_2_, and 5 mM iodoacetamide. Acetoacetyl-CoA produced from the assay was measured spectrophotometrically at 313 nm. OXCT1 activity was normalized to the total protein in the supernatants.

### Pull-down assay

Pull-down assay was performed as previously described (46). The following recombinant proteins were used: human full-length frataxin (precursor) fused to a C-terminal 6XHis tag (frataxin^1–210^-6XHis; Proteintech), intermediate (6XHis-frataxin^42–210^; Prospec, East Brunswick, NJ), and mature frataxin (frataxin^81–210^-6XHis; Dr Andrew Dancis, University of Pennsylvania).

### LC-MS analysis

Sample preparation: briefly, 20 μl serum was mixed with 50 μl 8 mM [^13^C_4_]-BHB and [^13^C_2_^2^H_3_]-acetate (in 80% methanol) before 400 μl of cold 80% methanol was added. The solution was homogenized by vortexing 5 s, then bath sonication in regular ice for 5 min. The solution was spun down at 17,000 *g* for 10 min at 4°C and the clear supernatant was collected. A volume of 5 μl supernatant was used for derivatization step. Liver, skeletal muscle tissue, and C2C12 cells were treated similarly.

Derivatization: derivatization reagent stocks were prepared fresh and used at final concentration of 1 mM in acetonitrile: 2-hydrazinoquinoline (HQ) 1 g/5 ml acetonitrile (1.256 M), 2,2-dipyridyl disulfide (DPDS) 22 mg/1 ml acetonitrile (0.1 M), and triphenylphosphine (TPP) 26.2 mg/1 ml acetonitrile (0.1 M). Derivatization mixture 1 (DM1) was made of 100 μl TPP stock, 100 μl DPDS stock and 5.8 ml acetonitrile. Derivatization mixture 2 (DM2) was made of 0.5 ml HQ stock and 4.5 ml of acetonitrile. In deep-well 96-well plate was combined 50 μl of DM1 and 5 μl of sample prepared as above. A volume of 50 μl of DM2 was added and the plate was incubated in water bath at 60°C for 15 mins. To stop the reaction, the plate was placed on ice for 5 mins to cool and 100 μl optima water was then added and vortex mixed. The plate was spin down at 3,000 rpm 4°C for 10 mins. A volume of 100 μl of the top (this was yellow in color) was transferred to HPLC plate. Calibration curves were constructed from water in the interval 11 μM to 0.01 μM using authentic standards as described previously (54).

LC-MS analysis: samples were analyzed using liquid-chromatography-high resolution mass spectrometry (LC-HRMS), with an Ultimate 3000 autosampler coupled to a Thermo Q-Exactive-HF instrument in positive electrospray ionization (ESI) mode scanning from m/z 70 to 950 and 120,000 resolution. LC separation was effectuated at 30°C on Acuity BEH C18 column 2.1 × 50 mm, 1.7 μm with solvent A: 2 mM ammonium acetate 0.05% acetic acid and solvent B: 2 mM ammonium acetate in 95% acetonitrile 0.05% acetic acid. Flow rate was 400 μl/min. The LC gradient was as follows: 0 min, 5% B; 2 min, 10% B; 5 min, 30% B; 6 min, 100% B; 8 min, 100% B; 8.6 min, 5% B; and 10.5 min, 5% B. Autosampler temperature was set at 5°C, and injection volume was 3 μl. Data processing was done with Xcalibur 4.0 software (Thermo Fisher Scientific Inc.).

### Statistical analysis

Statistical differences were analyzed by two-tailed Student’s t test and one way analysis of variance (ANOVA) test.

## Supplementary Material

pgac142_Supplemental_File

## Data Availability

All data is included in the manuscript and supporting information.
