# Supplementary material for: Frataxin controls ketone body metabolism through regulation of OXCT1
Source: PNAS Nexus. 2022 Jul 26;1(3):pgac142. doi: 10.1093/pnasnexus/pgac142 (PMC9396447; doi:10.1093/pnasnexus/pgac142)
Supplement: pgac142_Supplemental_File [file pgac142_supplemental_file.docx]

**Supplementary material for**

**Frataxin controls ketone body metabolism through regulation of OXCT1**

Yi NA Dong^1^, Clementina Mesaros^2^, Peining Xu^2^, Elizabeth Mercado-Ayon^3^, Sarah Halawani^1^, Lucie Ngaba^1^, Nathan Warren^1^, Patrick Sleiman^1^, Layne Rodden^1^, Kimberly A Schadt^1^, Ian A Blair^2^, David R Lynch^1, 3^

David R Lynch

Email: [lynchd@pennmedicine.upenn.edu](mailto:lynchd@pennmedicine.upenn.edu)

This PDF file includes:

Supplemental text

Table S1

Figure S1 to S8

Patient Histories

Patient 1: A 6 10/12 year-old boy presented to the hospital the day following arrival to the United States from Egypt with abdominal pain triggered by eating. Prior to admission he had a nearly 2-year history of intermittent abdominal pain, diarrhea, and failure to thrive. His parents were second cousins of Sudanese descent, and he had a 12-year-old brother in Egypt with a history of progressive ataxia. During his hospital stay he was noted to have failure to thrive (BMI 12.6 kg/m^2^) and chest pain following oral food intake; the chest pain resolved with G-tube placement and enteric feeds of an elemental diet. Echocardiogram identified moderate concentric left ventricular hypertrophy with a low normal ejection fraction, and 24-hour Holter monitor was normal despite ongoing episodes of chest pain, which were ascribed to vomiting or esophageal spasms.

Despite supplementation with fluids containing carbohydrates, he was persistently ketotic (initial beta hydroxy butyrate 4.5 mg/dl), with normal glucose and insulin levels and no evidence of insulin resistance. Leptin and triglycerides were also normal. On examination he had truncal and gait ataxia consistent with Friedreich ataxia. Long-range PCR analysis of intron 1 in *FXN* revealed two pathogenic GAA triplet repeats of 866 and 1266. SNP-based microarray identified various regions of homozygosity including the FXN gene on chromosome 9 and the *BSCL2* gene on chromosome 11.

Following discharge, ketosis and chest pain diminished with an elemental diet though recurrence was generally prompted by increased oral intake of fatty foods. Since recurrent ketosis is not a known feature of FRDA, a second causative condition was suspected. Research-based whole exome sequencing (WES) was performed on lymphocytes obtained from the proband, his siblings, and parents using the Agilent NGS target enrichment system for the Illumina HiSeq at the Children’s Hospital of Philadelphia Center for Applied Genomics. Data were quality controlled and analyzed using a custom-built pipeline that incorporates BWA-mem v0.7.12 for alignment, Picard v1.97 for PCR duplication removal, GATK v2.6.5 for variant calling and ANNOVA R for variant annotation. On average, 97.4% of the exome was sequenced at a depth of at least 20x. The proband was shown to be a carrier of a biallelic homozygous missense ENST00000433053.1:p.[(Ala282Thr)];[(Ala282Thr)] in *BSCL2*. The variant was confirmed by Sanger sequencing; both parents and an unaffected sister were heterozygous carriers. This variant is classified as a VUS in four submissions to ClinVar (Allele ID: 461415). *In silico* analysis by Polyphen and SIFT predict this missense variant has a deleterious effect on protein structure/function, minor allele frequency in gnomAD is reported at 3.58x10^-5^ (13 heterozygous alleles out of 282728; gnomAD v2.1.1 accessed 1/31/2022).

Patient 2. The 15-year-old brother of patient 1 presented for evaluation 3 years later, shortly after immigrating to the United States. He had failure to thrive (BMI 11.8), moderate dysphagia, scoliosis, and truncal ataxia. He was unable to stand unassisted and had a longstanding history of intermittent chest pain provoked by eating high-fat foods. This diminished with administration of an elemental diet after evaluation. Echocardiogram revealed marked concentric LVH with normal systolic function. Genetic testing identified similar *FXN* expansions and homozygosity for the *BSLC2* mutation found in his brother.

**Figure S1.** Frataxin overexpression increases OXCT1 protein levels in HEK293 cells. Representative blots and bar graph demonstrate increased OXCT1 levels in frataxin-transfected cells compared with control cells (n=4). ***p*<0.01. Data was shown as mean ± SE (error bars).

**Figure S2.** OXCT1 levels are reduced in the cerebellum of frataxin knockdown mice induced with doxycycline for 18 weeks. Representative blots and bar graph demonstrate reduced frataxin and OXCT1 levels in the cerebellum of frataxin knockdown mice compared with WT control (n=6). **p*<0.05, ***p*<0.01. Data was shown as mean ± SE (error bars).

**Figure S3.** PDH alpha subunit levels are unchanged in the cerebellum of KIKO mice. No change in PDH alpha subunit levels was found in the cerebellum of KIKO mice at either time point (n=4-7 per time point, *p*>0.05). Data was shown as mean ± SE (error bars).

**Figure S4.** Mitochondrial copy number is unchanged in the cerebellum of 12M KIKO mice. qPCR was performed to measure gene expression. mtDNA/nDNA (Mt-ND1/Cftr) ratio was unchanged in KIKO compared with control mice (A) (n=9-10). Similarly, mt-CO1 (B) and COX7A (C) mRNA levels were unchanged in KIKO compared with control mice (n=9-10). Data was shown as mean ± SE (error bars).

**Figure S5.** ATP5A levels are unchanged in the Purkinje neurons of cerebellar cortex of KIKO mice. While frataxin immunoreactivity is significantly reduced in KIKO mice (B), no change in ATP5A immunoreactivity was found in KIKO compared with control mice (A). Western blot analysis also showed unchanged ATP5A protein in the cerebellar homogenates of KIKO mice at 12M of age (C, D). GL, granular layer; ML, molecular layer; PL, Purkinje layer. Data was shown as mean ± SE (error bars).

**Figure S6.** Frataxin overexpression has no effect on the degradation of seipin. Representative blots and bar graph demonstrate reduced seipin protein levels over time in the presence of cycloheximide with or without frataxin in HEK293 cells (n=6). **p*<0.05, ^#^*p*<0.05, ** *p*<0.01, ^##^*p*<0.01. Data was shown as mean ± SE (error bars).

**Figure S7.** Thiolase levels are unchanged in the cerebellar homogenates of KIKO mice. While frataxin levels were significantly decreased in the cerebellar homogenates of KIKO mice at 12 month of age, no change was detected for thiolase compared with control (n=6-8). **p*<0.05. Data was shown as mean ± SE (error bars).

**Figure S8.** OXCT1 levels are decreased in the fibroblasts of FRDA patients. Patient1 shows similar OXCT1 levels as other FRDA patients (n=3 for control and n=4 for FRDA patients). ***p*<0.01. Data was shown as mean ± SE (error bars).
